# Therapeutic Application of Cardiac Stem Cells and Other Cell Types

**DOI:** 10.1155/2013/736815

**Published:** 2013-06-26

**Authors:** Emiko Hayashi, Toru Hosoda

**Affiliations:** Tokai University Institute of Innovative Science and Technology, 143 Shimokasuya, Isehara, Kanagawa 259-1193, Japan

## Abstract

Various researches on regenerative medicine were carried out experimentally, and selected modalities have been introduced to the clinical arena. Meanwhile, the presence of resident stem cells in the heart and their role in physiological cell turnover were demonstrated. So far skeletal myoblasts, bone marrow-derived cells, mesenchymal stromal cells, and resident cardiac cells have been applied for therapeutic myocardial regeneration. Among them, autologous transplantation of c-kit-positive cardiac stem cells in congestive heart failure patients resulted in an outstanding outcome, with long-lasting beneficial effects without major adverse events. By reviewing these clinical trials, an endeavor was made to seek for an ideal cellular therapy for cardiovascular diseases.

## 1. Introduction

Previously, somatic stem cells had been identified in multiple organs except for the heart, and attempts were made to utilize them for therapeutic regeneration of cardiovascular diseases; in particular, skeletal myoblasts and bone marrow-derived cells were employed in many clinical trials as discussed below. The heart was long viewed as a postmitotic terminally differentiated organ, in which the number of cardiomyocytes at birth is constant throughout the lifespan of the organism. In 2003, however, the presence of resident cardiac stem cell was reported [1], and the shift in paradigm occurred. More recently, the continuous turnover of myocardium in physiological and pathophysiological conditions has been demonstrated [2–4], improving dramatically our understanding of the self-renewing characteristic of the heart. This monumental discovery not only challenged the biological dogma but also opened a new path leading to an unprecedented therapeutic option for devastating cardiovascular diseases.

## 2. Skeletal Myoblasts

Skeletal myoblasts are undifferentiated cells separated from the muscular tissue. Although this cell type does not differentiate into myocardium, it is equipped with advantageous features with low tumorigenicity and relative tolerance for ischemia in contrast to cardiomyocytes. Based on preclinical studies showing improved cardiac function of infarcted myocardium after transplantation of skeletal myoblasts, the first clinical trial was initiated in 2001 [5] and later followed by others [6]. In these attempts, as many as hundreds of millions of cells were autologously transplanted, and some of the trials suggested the augmented systolic function with improved symptoms as the clinical outcome of the regimen. The beneficial effects of attenuating left ventricular remodeling were attributed to physical support, prevention of unfavorable disruption of extracellular matrix, and/or the paracrine mechanism through production and secretion of multiple growth factors.

The muscle cells differentiated from engrafted myoblasts are unable to form electrical coupling with surrounding cardiomyocytes and thereby failed to achieve integration and synchronicity with the host tissue, which was responsible for the refractory ventricular tachyarrhythmia observed in treated patients. For this reason, in the randomized double-blinded placebo-controlled MAGIC trial, all subjects received an implantable cardioverter defibrillator (ICD) prior to myoblast injection. However, the cardiac function did not improve with the treatment in the study [7].

Recently in Japan, a patient with dilated cardiomyopathy (DCM) was transplanted with autologous myoblast sheets manufactured utilizing temperature-responsive culture dishes; although this procedure involves thoracotomy, it may form a new therapeutic modality for heart failure patients [8].

## 3. Bone Marrow-Derived Hematopoietic Cells, Endothelial Progenitor Cells

Led by an epoch-making research on myocardial regeneration using c-kit-positive bone marrow cells [10], clinical usage of hematopoietic cells or endothelial progenitor cells for cardiac diseases has been continued since 2002 [11, 12]. As early as 2006, clinical trials employing more than 100 subjects were reported [13]. These cell types, unlike skeletal myoblasts, do not require a culturing process and are able to target an acute phase of the disease.

According to the recent meta-analysis of bone marrow cell therapy for acute and chronic ischemic heart failure, 36 randomized controlled trials and 14 cohort studies, involving, respectively, 996 and 464 treated patients, were performed worldwide; while the control groups consisted of 1,165 subjects, a total of 1,460 patients received autologous cells. The number of the cells transplanted was between 2 million and 60 billion (median 100 million), and, with an observational period of approximately six months, the left ventricular ejection fraction (LVEF) improved on average by 3.96% (95% confidence interval 2.90~5.02%) compared to that of the control subjects [14]. Particularly, patients with LVEF lower than 43% prior to cell therapy experienced a greater gain than those with preserved systolic function. Also, the analysis revealed that at least 40 million cells were necessary to provide any advantageous consequence [14]. Although it was unclear whether these beneficial effects were temporary in nature, many studies showed positive outcomes, and the meta-analysis indicated a reduction in the mortality as well as in the recurrence of myocardial infarction. Furthermore, the frequency of major adverse events, such as ventricular arrhythmias, did not significantly increase with the intervention [14].

There was a large deviation among the results of the trials, which may have been caused by various factors. When the meta-analysis was limited to the studies utilizing CD34-positive and/or CD133-positive cells, the significance in the improvement was lost [14]. However, in addition to the limited sample size of the subgroups, potential divergence in cell quality caused by sorting/preparation steps might lead to distinct conclusions. In fact, another meta-analysis suggested beneficial therapeutic effects of CD34-positive cells in a dose-dependent manner [15]. Also, the cell preparation methods and the quality control processes should affect the outcomes. In this regard, the subanalysis showed greater improvements in ventricular function with the use of heparin in the final cell suspension. On the contrary, however, a recent report documented that heparin impairs the homing capacity of bone marrow cells by blocking the interaction between SDF-1 and its receptor, CXCR4 [16]. These issues and others, such as cell storage conditions, are still unsolved, and further basic and/or clinical investigations are required.

Each study included in the meta-analysis had a relatively small number of subjects, employed a distinct observational period, and rarely designated mortality as its clinical endpoint. In order to overcome these limitations, the BAMI trial has been launched in 2012 as a collaborative effort of 11 European countries; this phase III open-label randomized controlled study, aiming at patients suffering from acute myocardial infarct, is testing the overall mortality and cardiac function, potentially influenced by coronary infusion of autologous bone marrow mononuclear cells (ClinicalTrials.gov NCT01569178). As many as 3,000 patients will be enrolled and followed for three years, and the study is expected to conclude in 2017. Additionally, bone marrow cells were assessed for their safety and efficacy in some clinical trials, as a therapeutic option for nonischemic heart failure, and appeared to be useful in this scenario [17, 18].

## 4. Mesenchymal Stromal Cells

Mesenchymal stromal cells (MSCs) are able to differentiate into osteoblasts, chondrocytes, and adipocytes in addition to myocytes and can be easily separated from other cells, by virtue of their tendency to adhere to plastic surface. It was also demonstrated that this cell type is equipped with a potential to give rise to cardiac myocytes and vascular endothelial cells, and its clinical application for the cardiovascular field was introduced in 2004 [21]. Currently many clinical trials targeting acute and chronic ischemic heart failure are ongoing; to date, they did not indicate concerns of major adverse events, at least in a short term, and proposed a promising trend towards improved symptom and ventricular function accompanying the treatment [22].

One of the attractive features of MSCs is the lack of major histocompatibility complex (MHC) class II on the cell surface and, therefore, relative tolerance against immunological reaction. If allogeneic transplantation becomes available, in theory prequalified potent cells could be expanded and stored for “off-the-shelf” usage whenever necessary. In this regard, Hare et al. reported that intravenous administration of allogeneic bone marrow MSCs resulted in improved cardiac function, coupled with reduced arrhythmic events of patients with acute infarct [23]. Moreover, the group subsequently demonstrated that catheter-based endomyocardial injections of autologous and allogeneic bone marrow MSCs, ranging from 20 million to 200 million in cell number, were similarly effective as the intervention against ischemic heart failure [24]; these manifestations raised the enthusiasm for allogeneic transplantation. However, upon differentiation of engrafted MSCs *in vivo*, they may acquire immunogenicity and could be eliminated by the immune system of the recipient [25], questioning the persistent benefit in the long run. The therapeutic effects of autologous and allogeneic MSCs on nonischemic heart failure are also under investigation in the POSEIDON-DCM trial (NCT01392625).

As for the mechanism of myocardial regeneration exerted by MSCs, in addition to their direct transdifferentiation into cardiac cell lineages, paracrine actions via secretion of a number of cytokines appeared to play a major role; these factors are shown to stimulate and activate c-kit-positive resident cardiac stem cells (CSCs), described in the next section, to accomplish the tissue restoration indirectly [26]. Another preclinical work implied synergistic regenerative effect of bone marrow MSCs injected together with CSCs [27], leading to the plan of the AIRMID trial, in which idiopathic dilated cardiomyopathy patients are scheduled to receive autologous transplantation of a mixture of these two cell types. Besides bone marrow-derived cells, MSCs of adipose tissue origin were lately applied clinically for heart failure patients, and some results have been disclosed [28].

## 5. Resident Cardiac Cells

**Figure 1:**
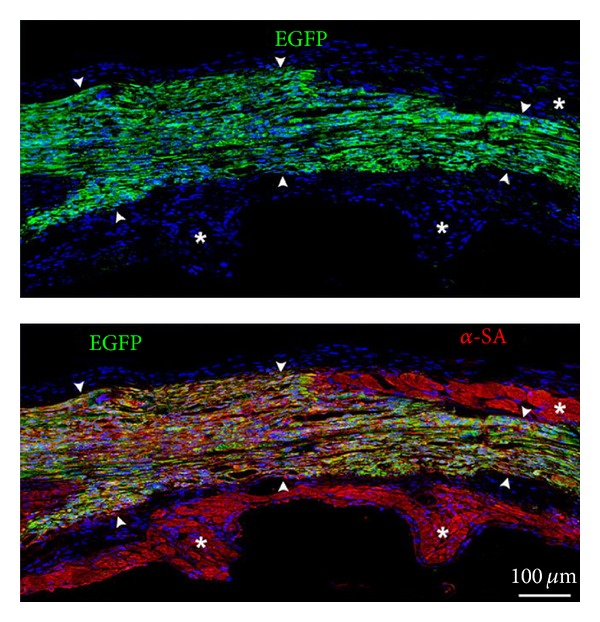
Regenerated myocardium formed by c-kit-positive human cardiac stem cells. Three weeks after injection of c-kit-positive human cardiac stem cells, labeled with enhanced green fluorescent protein (EGFP), into the border zone of the infarcted rat heart. Regenerated human cardiomyocytes (arrowheads) expressed EGFP together with alpha-sarcomeric actin (*α*-SA) and are distinguished from surviving rat myocytes possessing *α*-SA only (asterisks) [9].

**Figure 2:**
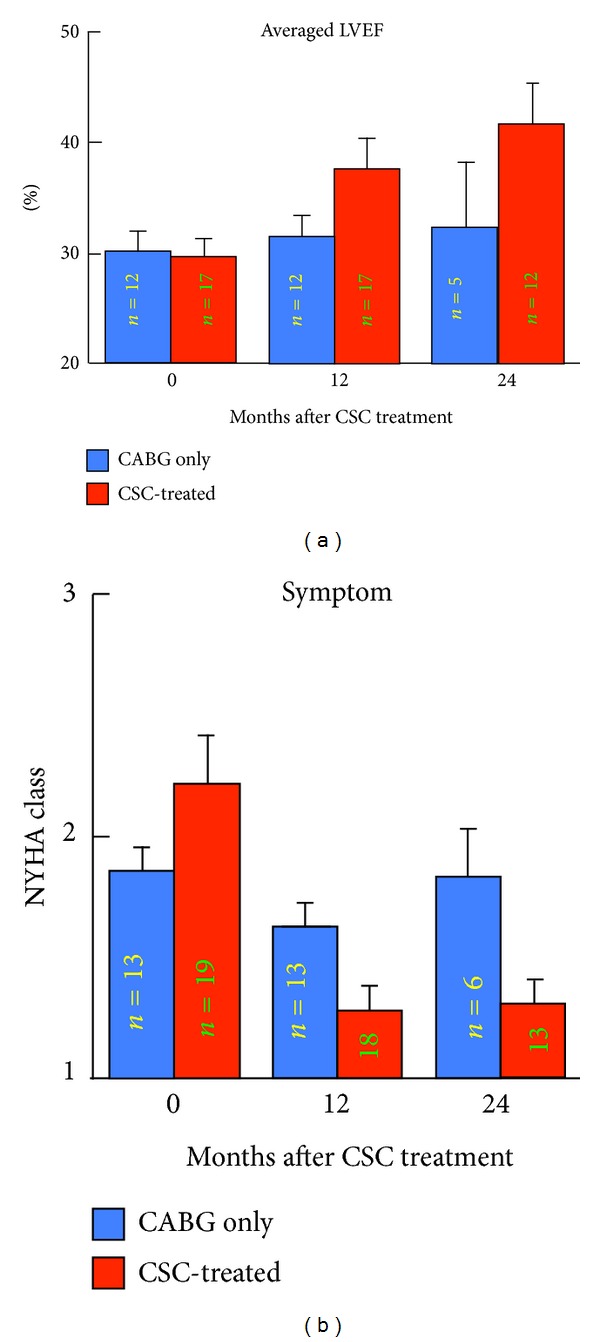
The SCIPIO trial targeting chronic heart failure. Autologous c-kit-positive cardiac stem cells (CSCs) were infused to the bypass graft of postinfarct patients. The trends of the left ventricular ejection fraction (LVEF) and the symptom categorized by the New York Heart Association (NYHA) functional classification are illustrated. Therapeutic effects lasted for more than two years without major adverse events. *n*: number of patients [19, 20].

As pointed in the beginning of this paper, growing evidence supported the notion that the homeostasis of the heart is maintained through continuous renewal of parenchymal cells, and c-kit-positive resident CSCs were identified in the human heart [30]. As depicted in Figure 1, the injection of human CSCs into the region bordering infarct in immunosuppressed rats resulted in the formation of regenerated human myocardium, accompanied by augmented cardiac performance. Consequently in 2009, the first clinical trial utilizing autologous c-kit-positive CSCs was initiated; this SCIPIO trial was an open-label randomized controlled study, aiming at post infarct patients undergoing an elective coronary artery bypass graft (CABG) surgery. The right atrial appendage, resected during the operation, was used to isolate and expand CSCs *in vitro*. Four months after CABG, only those with LVEF worse than 40% were eligible for the study, and, in the treated group, 1 million autologous CSCs were infused to the graft vessel via a balloon catheter. All 20 patients in the treated group successfully got transplanted with their own CSCs. As shown in Figure 2, while cardiac function of the control group basically did not change, the averaged LVEF of the treated patients improved from 30% at the baseline to 38% after one year and to 42% after two years of the cell infusion; the infarct size assessed by MRI decreased, and the symptom improved (Figure 2) in the CSC-treated group as well [19, 20]. Most importantly, this intervention did not increase the major adverse cardiac event rate [31]. The number of the subjects is small, but this sensational outcome surely warrants further investigation.

In clinical settings, for safety's sake it is uneasy to label cells for long-term tracking of the behavior following injection. Based on animal studies, c-kit-positive human CSCs are able to create, within one month after administration, even more human cardiomyocytes than the host myocytes lost due to the organ damage. Assuming a similar growth property of autologously transplanted CSCs, it is reasonable to interpret that the therapeutic effect, lasting for more than two years in the SCIPIO trial, was not only owing to the multiplication of the regenerated myocytes but also attributable to the maturation of each differentiated cell: namely, “cellular reverse remodeling”.

At the same time, cardiac cells capable of forming spheroid, that is, cardiosphere-derived cells (CDCs), were experimentally used in clinical studies. The CADUCEUS trial targeted patients experienced myocardial infarction within two to four weeks. CDCs were obtained from endomyocardial biopsy specimens and then cultured. Seventeen subjects, randomly assigned to the treated arm, were administered up to 25 million of CDCs through the culprit vessel, at one to two months after the biopsy; they were compared with the reference group consisting of eight patients. As demonstrated in the results, cardiac MRI showed an 8.4-gram reduction of the infarct size at six months and 12.9 grams at 12 months [32]. The global LVEF of the treated patients did not differ from that of the control subjects. However, it is noteworthy that the rate of adverse events did not significantly increase with this treatment.

Additionally, two Japanese research groups independently utilized CDCs for recent clinical studies; in the ALCADIA trial, six patients of ischemic heart failure received concomitantly CABG surgery, autologous CDC injection, and implantation of biodegradable gelatin-hydrogel releasing basic fibroblast growth factor (bFGF) (NCT00981006). Seven pediatric patients with a single ventricular chamber have been treated with autologous CDCs in the TICAP trial, and the results are awaited (NCT01273857).

## 6. Aiming for Optimized Cellular Therapy

**Figure 3:**
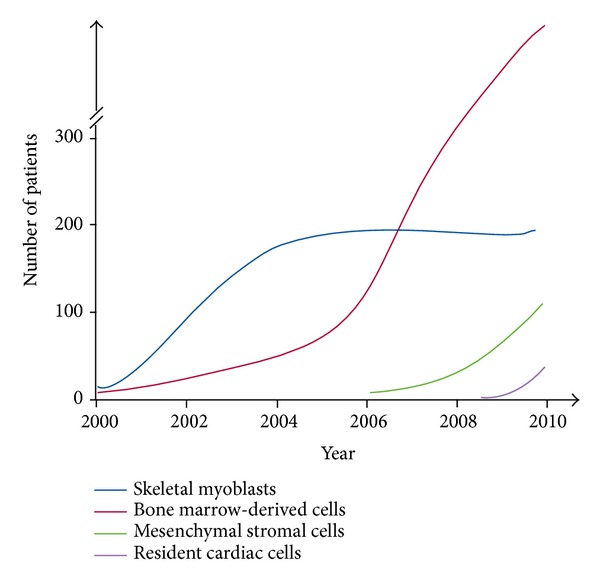
Progress of cellular therapies on ischemic heart diseases. The cumulative number of patients treated by each cell category is plotted [29].

As schematized in Figure 3 [29], clinical trials were initiated with skeletal myoblasts and followed by those using bone marrow-derived cells, whose enrolment is increasing remarkably. Subsequently, MSCs and resident cardiac cells emerged in the clinical arena with promising outcomes, and their usage would increase hereafter. Cellular therapy is generally inexpensive and would not cause immunological or ethical concerns, as long as autologous cells are used; the treatment could be repeated as required, especially, if low-invasive delivery methods are selected.

Here, we would like to list four major factors to be considered towards the optimization of the treatment. First, regarding the target population, the severer the diseases are, the more benefits the patients tend to take from the therapy; if the LVEF decreased only mildly, the patient would not be able to expect much gain from the intervention. Furthermore, it is appropriate to limit the application of an unestablished therapy to the sickest population at the beginning.

Secondly, as for the timing of the treatment, according to the subanalysis of the REPAIR-AMI trial, bone marrow cells, administered after five or more days of the onset of myocardial infarction, worked better than those injected at earlier time points [13]. This data may somewhat oppose our prediction but probably reflected the harsh environment for the exogenous cells to survive and engraft, shortly after the infarct. On the other hand, thanks to the progress of emergency medicine and intensive care, more patients with infarct are successfully rescued at the acute phase but may suffer from congestive heart failure at the chronic phase. Therefore, the demand on therapeutic approaches for chronic heart failure, of ischemic and nonischemic origin, will inevitably increase dramatically. Fortunately, cells requiring culturing maneuver, such as resident cardiac cells, could become an option in this context.

Thirdly, there are different delivery methods. Direct injection coupled with thoracotomy could be reasonably accomplished if combined with other surgical procedures; however, repetitive treatment using this modality would be unrealistic. As other possibilities, catheter-based intravascular infusion and intramyocardial injection were directly compared recently in a murine model. When c-kit-positive CSCs were administered two days after the infarct, intracoronary infusion resulted in uniform distribution of the cells, yielding a better clinical outcome than intramyocardial injection [33]. We could logically speculate that the corresponding difference in large animals or humans should be even greater; considering its low invasiveness and the possibility of repetition, the intracoronary route would be the best approach whenever applicable.

The last factor would be most important: the cell type to be used. Needless to say, the safety should be highly prioritized. In this regard, skeletal myoblasts, which may cause fatal arrhythmias, would be an unlikely choice. Also, although not yet applied clinically, induced pluripotent stem (iPS) cells would be questionable due to their tumorigenic potential. For extracardiac cells to contribute directly to myocardial regeneration, transdifferentiation through nuclear reprogramming is fundamental; this process, however, would take time and work with low efficiency. Also, the persistence of the acquired phenotype is uncertain. Instead, resident CSCs are destined to differentiate into the components of the heart, which makes it sensible to use them for this therapeutic purpose over pluripotent cells and those from extracardiac origin.

On the other hand, endomyocardial biopsy or other procedures for harvesting cardiac cells are more invasive than approaches such as bone marrow aspiration. Additionally, cardiac cells necessitate expansion prior to administration, which essentially precludes the autologous treatment at the acute phase of the first attack. However, this culturing step may actually function to select cells equipped with good property; since highly proliferative “juvenile” cells grow fast and dominate “senile” ones, after a while the culture dishes would be occupied solely by the healthy young population, which will be used for the treatment. This selection process might have assisted the efficacy with the relatively small deviations in the SCIPIO trial [19, 20].

Along with the cell types, the cell number is a related factor to be determined. Given a similar effectiveness, a smaller cell number would be favorable in view of potential adverse reactions. When plenty of cells are infused into a well-packed tissue like the heart, the consequent damage would be unignorable. In a recent study using immunosuppressed swine, 200 million human MSCs and one million c-kit-positive human CSCs, injected respectively to two-week-old infarct, showed similar clinical outcomes [27]. It is of note that the latter has revealed a powerful regenerative capability in the clinical setting as well [19, 20]. 

## 7. Conclusion

As discussed above, cellular therapy could be the last resort for heart failure. Its relatively low cost may contribute to health economics too. For acute heart failure patients, especially after myocardial infarct, bone marrow-derived cells would be the choice by virtue of their immediate availability. Instead, various modalities, with or without culturing procedures, are applicable for chronic heart failure. Therefore, clinical trials are needed to compare different cell types, to examine long-term outcomes, and to establish the safety and efficacy of repetitive treatment. In order to offer this advantageous technology to the general population as early as possible, supports from governments and/or public institutions are eagerly expected.
